# Analysis on fluid intake and urination behaviors among the elderly in five cities in China: a cross-sectional study

**DOI:** 10.3389/fnut.2023.1280098

**Published:** 2024-01-05

**Authors:** Yongye Song, Yue Zhang, Yan Liu, Jianfen Zhang, Junbo Lu, Xing Wang, Na Zhang, Guansheng Ma

**Affiliations:** ^1^Department of Nutrition and Food Hygiene, School of Public Health, Peking University, Beijing, China; ^2^YIDO AI Technology (Shandong) Co., Ltd., Jinan, China; ^3^Laboratory of Toxicological Research and Risk Assessment for Food Safety, Peking University, Beijing, China

**Keywords:** the elderly, fluid intake, urination behaviors, hydration status, cross-sectional study

## Abstract

**Background:**

Fluid intake in the elderly may influence urination behaviors and further influence their health status. This study investigated the behaviors of fluid intake, urination and their relationships among the elderly in China.

**Methods:**

Stratified random sampling was used to recruit the elderly participants who met the inclusion criteria from five cities in China. Participants’ total fluid intake (TFI) level was investigated using a validated 7-day 24 h fluid intake questionnaire. Their urination behaviors in real time were also recorded using a validated 7-day 24 h urination behavior record.

**Results:**

A total of 524 participants completed the study, including 233 males and 291 females. The average age was 69.7 years. The median daily TFI was 1,241 mL, with a frequency of 8.1 times per day. Approximately 73.3% of the participants did not reach the amount of adequate fluid intake (1.7 L for males and 1.5 L for females) recommended in China. Fluid intake in the morning, afternoon, and evening among participants was 594 mL, 305 mL and 342 mL, with a frequency of 3.0 times, 1.7 times, and 2.0 times, respectively. The median urination frequency was 7.4 times per day. The percentage of participants who urinated >7 times during the day was 44.3%. The percentage of participants who urinated ≥1 time at night was 77.5%. Age and BMI were not the main influence factors for fluid intake and urination behaviors. The preliminary analysis showed that higher TFI, plain water intake, dairy products intake, and fluid intake frequency were significantly associated with higher urination frequency (*t* = 6.553, *p* < 0.05; *t* = 5.291, *p* < 0.05; *t* = 4.667, p < 0.05; *t* = 13.413, *p* < 0.05). Higher fluid intake per time was significantly associated with lower urination frequency (*t* = −3.562, *p* < 0.05). Correlations between TFI, fluid intake frequency, fluid intake in night, fluid intake frequency in night and urination at night were also found (*r* = 0.114, *p* < 0.05; *r* = 0.091, *p* < 0.05; *r* = 0.146, *p* < 0.05; *r* = 0.331, *p* < 0.05).

**Conclusion:**

Fluid intake was inadequate in terms of the elderly participants. Participants with higher fluid intake and frequency in night had a greater incidence of nocturia. Thus, correcting fluid intake behaviors can improve urination behavior and promote health.

**Clinical trial registration:**

https://www.chictr.org.cn/searchprojEN.html, identifier CTR1900023355.

## 1 Introduction

Water is the main component of the human body, accounting for 60 ~ 70% of the total body mass (1). It is one of the most important nutrients for the maintenance of health and life (2). Water acts as a medium to support numerous metabolic functions: lubricating organs and transporting nutrients, hormones, and heat (3). The three water input sources include fluid intake from water and beverages, water intake from food, and endogenous water. The four pathways of water output include loss through urine through the urinary system, sweat through the surface of the skin, breath the through respiratory system, and feces through the digestive system (3, 4). There exists a balance between water input and water output. The two main sources of water input are fluid intake in the form of water and beverages and water from food. Similarly, urination accounts for about 60% of water output. It is widely recognized that proper hydration status is crucial for normal physiological function maintenance, including nutrient transport, excretion and body temperature regulation (5, 6). Recent research has revealed that insufficient fluid intake and dehydration can result in reduced physical and cognitive performances in the short term (7–10). In the long term, dehydration proved to be associated with the risk of chronic kidney disease, lithiasis, diabetes, and cardiovascular disease (11–14).

With aging, many physical functions decline to varying degrees in the elderly (15). The decline in digestion function may affect the absorption of water from food (16). Renal function declines, as a result, their glomerular filtration rate (GFR) and renal tubular function in terms of concentration, dilution, and transport decrease (17). Therefore, the proportion of water output from different sources may change (18). The body mass of the elderly undergoes changes due to the fact that the muscle mass decreases around the age of 40, leading to a gradual decrease in the overall water content of the body (19). The body water content of an elderly male individual accounts for approximately 47–67% of their body weight, while that of an elderly female individual accounts for approximately 39–57% of their body weight, which are lower than that of young adults (20–22). In addition, the physical activity level of the elderly reduces, leading to a decrease in the demand for energy (22), and, the energy consumption and metabolic rate of the elderly also decrease. According to the relationship between energy metabolism and water demand, the water demand of the elderly may be lower than that of young adults (23). In addition, the thirst center of the elderly decreases physiologically (24–26). As a result, they have a weaker sensation of thirst, while they do have the demand for fluid intake. This may lead to insufficient fluid intake among the elderly. Renal fluid conservation mechanisms were impaired among the elderly, leading to weakened responses to heat and cold stress (27, 28). Some other factors that may cause fluid restriction include urinary incontinence problems (29). A previous study has shown that fluid intake behaviors were influenced by various factors, including gender, age, body mass index (BMI), and environmental factors, such as temperature and humidity (30). Decreased fluid intake and increased water losses commonly cause dehydration in the elderly (6).

However, previous studies have shown that fluid intake in the elderly was less than that in younger individuals. A study conducted in Poland assessed water intake from food and beverages in 138 free-living elderly people aged 60 ~ 90 years old. The results showed that about 75% of elderly men and 51% of elderly women were under the recommended amount in terms of adequate water intake (including water intake from food and beverages; 2.5 L/d for elderly men and 2 L/d for elderly women aged 51 ~ 75 years and older) in Poland (2). Researchers assessed the total fluid intake (TFI) of 4,020 independent, community-living elderly people aged 65 years and above in Germany, which showed that the TFI level of elderly men was 700 mL, while that of women was 600 mL. The proportion of the elderly individuals aged 65–74 years who did not reach the recommended amount of adequate fluid intake (1,310 mL/d) in Germany was 44% of those aged 75 to 84 and 51% of those aged 85 and above (31). A study carried out in America using 24-h dietary recall data indicated that the TFI level (including plain water and moisture in beverages) for the elderly (≥60 years old) was 2071 mL, which was lower than the levels of adults aged 20 ~ 39 years (2,736 mL) and 40 ~ 59 years (2,797 mL) (32). The recommended amount of adequate fluid intake for elderly individuals in China is the same as adults: 1.7 L/d for males and 1.5 L/d for females, with a total water intake level of 3.0 L/d and 2.7 L/d, respectively (33). A study conducted in 2012 investigated daily TFI and dietary consumption status among 413 participants aged 50 ~ 75 years with chronic diseases in China. The results found that the average daily TFI level was 950 mL/d, as 55% of the participants did not meet fluid intake recommendations (1.2 L/d for the elderly according to Chinese Dietary Reference Intakes 2007) (34, 35). The values set at of 2007 were much lower than the currently recommended amount of adequate fluid intake (1.7 L/d for males and 1.5 L/d for females). Data from China Nutrition and Health Surveys (2015 ~ 2017) showed that the total intake of plain water and tea for the elderly aged 60 ~ 74 years was 846 mL/d (36, 37). Thus, it can be seen that few studies have provided data on fluid intake for the elderly and that the phenomena of insufficient fluid intake is widespread among the global elderly population. Moreover, there is a lack of in-depth analysis on the frequency and types of fluid intake in relation to elderly individuals (38). Besides fluid intake from water and beverages, food is another main source of water. However, residents in European and American countries consume about 20% of their daily water intake from food, while residents in China can reach 40–49% (39, 40). Although the data from other countries can provide references as a basis for fluid intake behaviors of the elderly in China, it cannot be used to determine the adequate water intake for Chinese people. Research on elderly people aged more than 65 years in China is needed.

Urine is the main pathway for water excretion, accounting for approximately 60% of total daily water excretion. Declining kidney function weakens fluid balance regulation (20, 22, 41). Urination behaviors refer to the behaviors of the body’s excretion of urine after sensing the volume of urine in the bladder (42). These behaviors relate to the volume of urine being excreted, the frequency of urination, urinary urgency, and place of urination (43, 44). Urine indicators and urination behaviors can be used to determine hydration status (45–47). A decrease in urine volume indicates a risk of dehydration (48). Unhealthy urination behaviors also can be risk factors for urinary system diseases (44). A study conducted on 636 female nurses indicated an association between unhealthy toileting behaviors and susceptibility to lower urinary tract symptoms (49). Increased urination promotes bacteria elimination from the urinary tract and bacterial proliferation reduction in the bladder (50–52). Previous studies have explored the urination behaviors among Chinese women and students in different stages (53–55), while few studies focused on the elderly. Moreover, the association between fluid intake behaviors and urination behaviors remains unclear. A survey of European adults has shown that individuals with higher water intake levels tended to have higher urine volume (56). Another study conducted on 87 young adults evaluated urination number within 24 h as a possible indicator of hydration status. The results indicated that dehydrated individuals had higher 24 h urine volume (1933 versus 967 mL), a great number of urination (five versus three times), lower urine specific gravity (1.012 versus 1.025) and lower urine osmolality (457 versus 874, mOsm/kg) (57). However, research on the relationship between fluid intake behaviors and urination behaviors among the elderly in China has not been carried out yet.

In this study, the primary objective was to investigate the fluid intake behaviors and urination behaviors of the elderly in China. The influence factors of age and BMI were also analyzed. Further, the associations between fluid intake behaviors and urination behaviors were analyzed. The results of the study can provide scientific reference data for fluid intake and urination behaviors for the elderly in China. Moreover, it contributes to possible strategies that can be used to promote healthy fluid intake and urination behaviors.

## 2 Methods

### 2.1 Study design

The survey was conducted from October 1 to October 31, 2019. Considering the influence of the relatively large geographical span of China, five cities from different geographical regions were selected in our present study, including Taiyuan, Jinan, Hefei, Nanchang, and Guangzhou. Jinan, Hefei, and Nanchang are located in Central China, while Jinan is located in the north, while Hefei and Nanchang are located in the south. Taiyuan is located in North China, and Guangzhou is located in South China. A stratified random sampling method was used to recruit the elderly. Participants from five cities in China were enrolled in this study.

### 2.2 Sample size calculation

In our study, the sample size (n) was calculated using the following formula: *n* = *t*^2^*P*(1–*P*)/*e*^2^ (58). In the formula, “*t*” represents the corresponding statistic value when the confidence was set as 95%, that is, *α* = 0.05 and *t* = 1.96. In addition, “e” represents the error and was set as 5%. One study conducted among the elderly in China revealed that the detection rate (P) of adults with a fluid intake under the recommended amount of adequate fluid intake was 32.4% (59). The prevalence of frequent or urgent urination found in 1164 patients, which was 24.3% (60). The calculated sample size was 337 when P was set as 32.4%; and the calculated sample size was 283 when P was 24.3%. Considering stratification (five cities) and a missed follow-up rate of 10%, at least 374 participants were required for the study to achieve validity.

### 2.3 Participants

In October 2019, recruitment notices targeting the elderly in these five cities were published online. The inclusion and exclusion criteria, as well as an introduction to the research, were displayed. The inclusion criteria were as follows: age range between 60 and 80 years; living or working in Jinan, Taiyuan, Hefei, Nanchang, or Guangzhou for 3 months or more before study; having a good mental state; and the capacity to fill in questionnaires independently. The exclusion criteria were as follows: diseases relating to cognitive dysfunction, schizophrenia, other mental disorders, chronic kidney disease, or urinary system diseases; damage to the nervous system; having a pacemaker or other embedded electronic medical devices; having physical activity disorders; receiving treatment involving a restricted diet or fluid intake; having a history of using any diuretics or related medications; and having a history or habit of excessive drinking. Registration information was collected using electronic questionnaires. After being included in the study, all participants signed informed consent forms.

### 2.4 Ethical standards

The study protocol was reviewed and approved by the Ethical Review Committee of Peking University. The ethical approval project identification code is IRB 00001052-18039. The study protocol was registered on the Chinese Clinical Trial Registry website under trial registration number Chi CTR 1900023355. The study was conducted in accordance with the principles of the Declaration of Helsinki. Prior to the beginning of the study, written informed consent forms were obtained from all the participants before the study voluntarily. The participants and the researchers each reserved one copy of the informed consent form.

### 2.5 Study procedure

Participants’ height and weight were measured after they were enrolled. After training by the researchers, fluid intake behaviors and urination behaviors were recorded for 7 consecutive days by the participants. From day 1 to day 7, their fluid intake behaviors were recorded using the 7-day 24 h fluid intake questionnaire in real time under free-living conditions (61). Participants’ TFI values were evaluated and recorded by themselves using a uniformly customized cup with a scale to the nearest 10 mL as a reference. However, the water intake level from food was not measured or recorded. Using the validated 7-day 24 h urination behavior record, the participants’ urination behaviors was recorded by themselves in real time, including frequency of urination, time of urination, and urinary urgency. During the study period, the researchers guided the participants to complete the questionnaire and took photos for verification. Participants’ completed questionnaires were checked by the researchers every day. The indicators collected during different study time points are presented in Table 1.

**Table 1:** The indicators collected at different time points in the study.

	Day 1	Day 2	Day 3	Day 4	Day 5	Day 6	Day 7
Individual information	√						
Anthropometric measurement	√						
7-day 24 h fluid intake questionnaire	√	√	√	√	√	√	√
7-day 24 h urination behavior record	√	√	√	√	√	√	√
Temperature	√	√	√	√	√	√	√

### 2.6 Anthropometric measurements

A height–weight meter (HDM-300, Huajun, Zhejiang, China) was used to measure participants’ height and weight under fasting status. The participants were dressed in light clothing and stood barefoot on the meter with their backs facing the scale, their torsos naturally straight, and their heads remaining upright throughout the process. The height and weight of the participants were measured twice to the nearest 0.1 cm and 0.1 kg, respectively. The averages for height and weight were calculated. Then, their BMI values were calculated: BMI (kg/m^2^) = weight (kg)/height (m) squared.

### 2.7 Measurement of daily total fluid intake behaviors

In our study, total water intake includes two parts, namely, fluid intake from water and beverages (accounting for 50% approximately) and water intake from food (accounting for 40% approximately) (22). Daily TFI refers to the amount of fluid intake from water and beverages, excluding water from food. A 7-day 24 h fluid intake questionnaire was used to record fluid intake behaviors, including daily TFI and patterns of fluid intake. This questionnaire has been validated and applied in a large body of research and practical work (62–64). It is an authoritative and commonly used questionnaire in different countries in the field of fluid intake. It has undergone consultation with experts and repeated argumentation, and expert consensus has been reached. It was based on the validated questionnaire used in previous studies, which was revised in line with the purpose of this survey (65, 66). After undergoing standardized training by the researchers, TFI levels were collected and recorded using the 7-day 24 h fluid intake questionnaire by the participants. A customized cup was used to measure the fluid intake level for each time period over the 7 consecutive days, whose scale was accurate to 10 mL. According to the General Standard for Beverages of China (GB/T 1-789-2015) and the methods used in previous studies, all the drinking fluids were classified into plain water, dairy products, tea, sugar-sweetened beverages, plant protein beverages, fruit/vegetable juices, and other beverages (67–69). The types of plain water included in the study referred to tap, packaged, or mineral and purified. The types of dairy products referred to pure milk, yogurt, and other dairy products without the addition of sugar in the production process. Sugar-sweetened beverages (SSBs) refer to beverages with the addition of sugar in the beverage production process, including carbonated, coffee, plant-based, flavored, protein, and special-purpose beverages. Plant protein beverages refer to products made using plant fruits, seeds, or kernels with a certain amount of protein as raw materials, with or without the addition of other food raw materials, including soymilk, soybean milk, soybean milk beverages, and coconut milk. The types of tea included self-made green, scented, and black, excluding tea beverages that include added sugar. Fruit and vegetable juice refers to beverages made from various fruits and vegetables through grinding, pressing, and other processes, excluding beverages with added sugar, fruit- and vegetable-flavored beverages. Beverages that do not belong to the above classification were classified as other beverages. The time and location of fluid intake behaviors were recorded by the participants. The records were captured and sent to the investigators using a mobile phone every day, which were then reviewed to ensure the accuracy and completeness of the fluid intake records.

Regarding time periods of fluid intake, the day was divided into eight time periods: before breakfast, during breakfast, after breakfast, during lunch, after lunch, during dinner, after dinner, and at night. The morning referred to “before breakfast,” “during breakfast,” and “after breakfast” periods, whereas the afternoon referred to “during lunch” and “after lunch” periods, while the evening referred to “during dinner,” “after dinner,” and “night” periods.

### 2.8 Measurement of urination behaviors

A 7-day 24 h urination behavior record was used to record the information regarding participants’ urination behaviors after training. The record was designed based on a previous questionnaire through expert consultation and was concerned with the frequency of urination, time of urination, and urinary urgency (43, 54).

The frequency of urination was recorded as the number of times the participant urinated per day, from the first urination of the day after waking up to the last urination before waking up the next day. The 7-day average frequency of urination was calculated from these numbers.

Regarding the time periods of urination, the day was divided into eight time periods: before breakfast, during breakfast, after breakfast, during lunch, after lunch, during dinner, after dinner, and at night. The morning referred to “before breakfast,” “during breakfast,” and “after breakfast” periods, whereas the afternoon referred to “during lunch” and “after lunch” periods, while the evening referred to “during dinner,” “after dinner,” and “night” periods.

According to the International Continence Society report on urinary function, frequent urination was defined as urinating more than 7 times during the day (70), and nocturia was defined as urinating more than 1 time at night (71).

### 2.9 Temperature

According to data from the China Meteorological Administration, the daily minimum and maximum temperatures in the five cities, including Taiyuan, Jinan, Hefei, Nanchang, and Guangzhou, during the survey period were recorded. The temperature of the day was defined as the median temperature, and the average temperature in each city during the study period was calculated.

### 2.10 Quality control

A unification procedure was established before the study. This survey was conducted after unified training for researchers and participants. Study guidance was developed for the study, including the research protocol, questionnaire and records, methods, and procedures. Effective and convenient methods of communication between the participants and investigators were used to improve the follow-up rate and quality of the data, such as home visits and telephone calls. The time and reasons for dropout were also recorded in detail. Throughout the study period, all the procedures were strictly supervised by researchers for quality control. Double-checking was performed on the completed questionnaires each day. Before data inputting, the researchers coded and checked each item in the questionnaire and cleared the error items.

### 2.11 Statistical analysis

The EpiData 3.1 software was used to establish the database, and data entry was carried out via the double-entry method to check and clean up the wrong items promptly. SPSS Statistics 26.0 (IBM Crop, Armok, NC, USA) was used to perform the statistical analysis. The participants were divided into four groups according to the quartile of their sex. The data were subjected to normality tests. The results were reported as mean ± standard deviation (SD) if the data were normally distributed. If not, then the median and quartile ranges (M and Q) were used to reveal the data. Differences in the normally distributed data (reported as mean ± SD), such as age, height, weight, and BMI, were compared using one-way ANOVA among different groups. Kruskal–Wallis rank-sum tests were used to compare the differences in the non-normally distributed data (shown as M and Q) among different groups. Count data were described as n (percentage), and a chi-square test was used to compare the differences in these indexes. The Student–Newman–Keuls (SNK) method (*p* < 0.05) was used to compare the differences evident between each of the two groups. Linear regression models were used to examine the relationships between urination frequency and fluid intake behaviors in 24 h. Spearman’s correlations were used to analyze the correlations between night urination frequency and fluid intake behaviors. Since urination in the night is a binary variable, point biserial correlations were used to analyze the correlations between fluid intake behaviore and urination at night. The significance level was set at 0.05 (*p* < 0.05).

## 3 Results

### 3.1 Participants’ characteristics and temperature

A total of 551 participants from five cities who met the inclusion criteria were enrolled in this study. No participant lost contact during the investigation period. Among them, 27 dropped out voluntarily due to incomplete data. After invalid questionnaires were eliminated, the data of 524 participants remained for analysis, with a completion rate of 95.1% (Table 2).

**Table 2:** Characteristic of the participants.

	Total			Males			Females		
	*n* (%)^a^	Age^b^	BMI^b^	*n* (%)^a^	Age^b^	BMI^b^	*n* (%)^a^	Age^b^	BMI^b^
Total	524	69.7 ± 5.3	26.3 ± 2.5	233 (44.5)	71.0 ± 5.4	26.2 ± 3.3	291 (55.5)	68.7 ± 5.1	26.3 ± 2.6
Taiyuan	93 (17.7)	68.4 ± 5.5^c^	26.6 ± 2.2	39 (41.9)	70.2 ± 5.5	26.8 ± 2.2^c^	54 (58.1)	67.1 ± 5.2^c^	26.4 ± 2.3
Jinan	96 (18.3)	69.3 ± 5.1^c^	26.5 ± 2.8	48 (50.0)	70.1 ± 5.3	26.1 ± 2.4^d^	48 (50.0)	68.5 ± 4.8^c^	27.0 ± 3.1
Hefei	121 (23.1)	71.0 ± 4.9^d^	26.3 ± 2.4	53 (43.8)	71.9 ± 5.3	26.2 ± 1.7^e^	68 (56.2)	70.3 ± 4.4^d^	26.4 ± 2.8
Nanchang	116 (22.1)	70.9 ± 4.6^d^	25.7 ± 2.1	54 (46.6)	71.6 ± 4.7	25.4 ± 2.0^d^	62 (53.4)	70.2 ± 4.5^d^	26.0 ± 2.1
Guangzhou	98 (18.7)	68.6 ± 6.0^c^	26.2 ± 2.7	39 (39.8)	70.8 ± 6.1	26.5 ± 3.0^d^	59 (60.2)	67.1 ± 5.6^c^	26.0 ± 2.6
*F*		5.965	2.286		1.112	2.663		6.252	1.233
*p*		<0.001^*^	0.059		0.351	0.033^*^		<0.001^*^	0.297

^a^Values presented as numbers (percentage). ^b^Values presented as mean ± SD and compared using one-way ANOVA. ^*^Means that there was a significant difference as the value of *p* was less than 0.05. *F* is the statistic in analysis of variance. It is the ratio of inter group mean square (MS inter group) and intra group mean square (MS intra group). ^c–e^The same symbol indicates that there were no statistically significant differences between the two groups; different symbols indicate that the differences were statistically significant between the two groups. SD, standard deviation; BMI, body mass index. BMI (kg/m^2^).

The participants included 233 men (44.5%) and 291 women (55.5%). Their average age was 69.7 years, which differed significantly between the participants from five cities (*F* = 5.965 *p* < 0.05). Their average BMI was 26.3 ± 2.5 kg/m^2^. Participants were divided into four groups according to the quartiles of their BMI (Q1: 17.8 ~ 24.4, Q2: 24.5 ~ 25.7, Q3: 25.8 ~ 27.7, Q4: 27.7 ~ 36.6) (Tables 3–5).

**Table 3:** Fluid intake patterns of the participants with different characteristics.

	Total						Males						Females					
	Daily TFI (mL)^a^	Percentage under Chinese water recommendation level (%)^b^	Plain water (mL)^a^	Tea (mL)^a^	Dairy products (mL)^a^	SSBs (mL)^a^	Daily TFI (mL)^a^	Percentage under Chinese water recommendation level (%)^b^	Plain water (mL)^a^	Tea (mL)^a^	Dairy products (mL)^a^	SSBs (mL)^a^	Daily TFI (mL)^a^	Percentage under Chinese water recommendation level (%)^b^	Plain water (mL)^a^	Tea (mL)^a^	Dairy products (mL)^a^	SSBs (mL)^a^
Total	1,241 (644)	73.3	868 (684)	43 (193)	193 (252)	0 (0)	1,229 (642)	79.0	819 (731)	86 (288)	120 (249)	0 (0)	1,253 (664)	68.7	900 (659)	20 (131)	160 (229)	0 (0)
*Z*							0.109	7.559	2.077	4.034	2.039	2.309						
*p*							0.913	0.006^#^	0.038^#^	0.000^#^	0.008^#^	0.021^#^						
Taiyuan	1401 (818)^c^	62.4	1049 (840)^c^	0 (144)^c^	217 (234)^c^	0 (0)^c^	1,401 (765)	71.8^c^	959 (910)	79 (244)	219 (267)	0 (0)	1,398 (876)	62.5^c^	1081 (856)^c^	0 (67)^c^	216 (231)	0 (0)^c^
Jinan	1219 (958)^d^	66.7	771 (818)^d^	114 (318)^d^	116 (216)^d^	0 (0)^c^	1,166 (973)	70.8^c^	702 (647)	157 (358)	60 (183)	0 (0)	1,281 (894)	55.6^d^	784 (978)^d^	89 (305)^d^	143 (233)	0 (0)^c^
Hefei	1164 (442)^d^	82.6	843 (444)^e^	50 (219)^c^	143 (198)^d^	0 (0)^c^	1,124 (481)	90.6^d^	740 (507)	0 (102)	159 (211)	0 (0)	1,170 (409)	58.1^d^	894 (366)^e^	29 (137)^e^	143 (184)	0 (0)^c^
Nanchang	1369 (904)^e^	63.8	937 (958)^f^	0 (0)^e^	107 (249)^e^	0 (0)^c^	1,300 (913)	70.4^c^	896 (946)	114 (399)	75 (168)	0 (0)	1,404 (845)	76.5^e^	996 (918)^f^	0 (0)^c^	161 (267)	0 (0)^c^
Guangzhou	1231 (344)^e^	89.8	840 (498)^g^	92 (158)^c^	157 (277)^e^	0 (15)^d^	1,230 (329)	92.3^d^	871 (643)	151 (157)	104 (250)	0 (0)	1,231 (321)	88.1^f^	833 (433)^e^	79 (144)^f^	203 (254)	0 (29)^d^
*H*	10.347	32.208	14.718	67.572	15.526	37.804	3.904	13.996	6.130	20.171	9.967	6.709	7.005	20.742	13.878	53.806	8.742	31.619
*p*	0.035^*^	<0.001^*^	0.005^*^	<0.001^*^	0.004^*^	<0.001^*^	0.419	0.007^*^	0.190	<0.001^*^	0.041^*^	0.152	0.136	<0.001^*^	0.008^*^	0.000^*^	0.068	<0.001^*^
Age (y)																		
≥60, <65	1,264 (588)	72.1	825 (751)	44 (174)	166 (224)	0 (0)^c^	1,221 (465)	84.2	805 (728)	95 (230)	104 (207)	0 (0)	1,286 (668)	65.2	831 (782)	32 (36)	188 (235)	0 (0)^c^
≥65, <70	1,229 (634)	71.8	871 (608)	57 (205)	131 (250)	0 (0)^c^	1,210 (753)	75.6	736 (674)	171 (464)	93 (269)	0 (0)	1,236 (633)	70.2	904 (571)	39 (142)	143 (219)	0 (0)^c^
≥70, <75	1,231 (764)	71.6	821 (823)	30 (204)	160 (271)	0 (0)^c^	1,266 (874)	73.2	780 (898)	64 (343)	144 (255)	0 (0)	1,194 (690)	69.9	887 (675)	0 (133)	164 (244)	0 (0)^c^
≥75, <80	1,260 (570)	78.4	919 (620)	36 (186)	138 (250)	0 (0)^d^	1,201 (553)	85.3	871 (620)	60 (222)	106 (249)	0 (0)	1,309 (663)	68.8	1,045 (642)	0 (84)	161 (267)	0 (0)^d^
*H*	2.039	2.039	1.991	1.016	3.73	10.692	1.111	4.242	1.917	3.398	1.597	5.867	1.630	0.540	2.413	1.794	5.301	8.325
*p*	0.564	0.564	0.574	0.797	0.292	0.014^*^	0.774	0.236	0.590	0.334	0.660	0.118	0.653	0.910	0.491	0.616	0.151	0.040^*^
BMI (kg/m^2^)																		
17.8 ~ 24.4	1,266 (526)	74.8	835 (576)	43 (164)	173 (279)^c^	0 (0)	1,314(493)	81.0	869 (628)	0 (145)^c^	151 (241)	0 (0)	1,230 (561)	72.0	826 (590)	49 (172)	189 (258)^c^	0 (0)
24.5 ~ 25.7	1,279 (643)	74.0	929 (754)	30 (177)	143 (279)^d^	0 (0)	1,240 (630)	81.4	803 (764)	92 (293)^d^	136 (279)	0 (0)	1,285 (692)	67.2	971 (703)	0 (76)	171 (240)^c^	0 (0)
25.8 ~ 27.7	1,297 (761)	68.7	876 (809)	44 (240)	130 (250)^d^	0 (0)	1,205 (854)	71.7	750 (756)	155 (342)^e^	94 (240)	0 (0)	1,404 (639)	61.8	1,096 (590)	0 (121)	143 (250)^d^	0 (0)
27.7 ~ 36.6	1,146 (700)	75.6	843 (668)	50 (194)	133 (243)^e^	0 (0)	1,137 (656)	82.1	789 (699)	93 (354)^d^	111 (248)	0 (0)	1,136 (724)	71.3	853 (664)	21 (126)	143 (218)^d^	0 (0)
*H*	1.949	1.949	0.878	0.87	8.784	2.341	1.781	2.618	2.915	9.138	1.689	6.164	2.058	1.955	3.381	5.506	8.625	1.320
*p*	0.583	0.583	0.831	0.833	0.032^*^	0.505	0.619	0.454	0.405	0.028^*^	0.639	0.104	0.560	0.582	0.337	0.138	0.035^*^	0.724

^a^Values presented as median (quartile ranges) and compared using the Kruskal–Wallis test. ^b^Values presented as percentage and compared using the Kruskal–Wallis test. The four groups of BMI were divided according to the quartile range of BMI. ^#^Means that there was significant difference between males and females. ^*^Means that there was significant difference among participants in different cities/age groups/BMI groups, as the value of *p* was less than 0.05. ^c–g^The same symbol indicates that there were no statistically significant differences between the two groups; different symbols indicate that the differences were statistically significant between the two groups. The recommendation for TFI levels for the male and female elderly set by the Chinese nutrition society are 1.7 and 1.5 L per day, respectively. BMI: body mass index (kg/m^2^); TFI, total fluid intake; SSBs, sugar-sweetened beverages.

**Table 4:** Daily fluid intake of participants with different characteristics in different time periods.

	Total						Males						Females					
	Morning		Afternoon		Evening		Morning		Afternoon		Evening		Morning		Afternoon		Evening	
	Fluid intake (mL)	Times	Fluid intake (mL)	Times	Fluid intake (mL)	Times	Fluid intake (mL)	Times	Fluid intake	Times	Fluid intake (mL)	Times	Fluid intake (mL)	Times	Fluid intake (mL)	Times	Fluid intake (mL)	Times
Total	594 (281)	3.0 (1.3)	305 (223)	1.7 (1.1)	342 (224)	2.0 (1.3)	584 (280)	3.0 (1.4)	301 (212)	1.7 (1.1)	350 (247)	2.0 (1.3)	600 (287)	3.0 (1.3)	307 (233)	1.7 (1.3)	329 (211)	2.0 (1.4)
*Z*							0.998	1.674	0.491	0.033	1.267	0.707						
*p*							0.318	0.094	0.623	0.974	0.205	0.480						
Cities																		
Taiyuan	679 (353)^b^	3.0 (1.4)^b^	357 (275)	1.6 (1.1)^a^	350 (248)	1.9 (1.5)^a^	669 (319)	3.0 (1.6)	326 (239)	1.6 (1.0)	374 (270)^a^	2.0 (1.3)	715 (388)^a^	3.2 (1.3)^a^	368 (338)	1.6 (1.1)	319 (228)	1.7 (1.6)
Jinan	568 (398)^a^	2.6 (1.3)^a^	301 (296)	1.4 (1.1)^b^	349 (283)	1.9 (1.3)^a^	536 (378)	2.6 (1.3)	301 (332)	1.5 (1.6)	359 (287)^a^	1.9 (1.0)	599 (416)^b^	2.6 (1.7)^b^	290 (328)	1.4 (1.1)	342 (296)	1.9 (1.3)
Hefei	540 (211)^c^	3.0 (1.0)^b^	300 (184)	1.7 (1.1)^a^	324 (178)	2.1 (1.1)^b^	559 (219)	3.0 (0.9)	291 (177)	1.7 (1.0)	311 (172)^b^	2.0 (1.2)	537 (216)^c^	3.0 (1.0)^c^	301 (189)	1.6 (1.1)	338 (174)	1.4 (2.1)
Nanchang	604 (372)^a^	3.0 (1.4)^b^	300 (296)	1.9 (1.5)^a^	371 (284)	2.1 (1.3)^b^	587 (376)	3.0 (0.9)	300 (286)	1.9 (1.2)	400 (292)^c^	2.2 (1.3)	629 (326)^d^	3.1 (1.4)^a^	296 (328)	1.9 (1.9)	339 (270)	2.0 (1.2)
Guangzhou	586 (154)^a^	3.4 (1.0)^b^	298 (150)	2.0 (1.1)^c^	304 (171)	2.1 (1.1)^b^	580 (164)	3.7 (1.0)	313 (160)	2.0 (1.1)	336 (187)^d^	2.3 (1.4)	587 (148)^e^	3.3 (0.9)^d^	296 (152)	1.9 (1.0)	299 (161)	2.1 (1.4)
*H*	14.540	37.028	8.524	17.953	9.451	12.435	4.167	1.984	2.321	1.780	9.792	0.803	11.503	16.125	7.358	8.847	3.835	5.980
*p*	0.006^*^	<0.001^*^	0.074	0.001^*^	0.051	0.014^*^	0.384	0.576	0.677	0.619	0.044^*^	0.849	0.021^*^	0.003^*^	0.118	0.065	0.429	0.201
Age (y)																		
≥60, <65	595 (275)	3.0 (1.3)	301 (250)	1.8 (1.3)	332 (200)	2.0 (1.1)	550 (264)	3.0 (1.4)	280 (160)	1.8 (0.9)	339 (253)	2.1 (1.3)	616 (281)	3.1 (1.3)	320 (264)	1.8 (1.6)	326 (189)	2.0 (1.3)
≥65, <70	593 (293)	3.0 (1.4)	308 (211)	1.7 (1.1)	333 (257)	2.0 (1.2)	613 (412)	3.0 (0.9)	300 (179)	1.6 (0.9)	343 (261)	2.0 (0.7)	590 (264)	3.0 (1.5)	308 (216)	1.7 (1.4)	327 (270)	2.0 (1.6)
≥70, <75	591 (301)	3.1 (1.1)	301 (257)	1.9 (1.3)	331 (239)	2.0 (1.1)	592 (263)	3.0 (1.5)	331 (287)	1.9 (1.3)	359 (320)	2.1 (1.3)	586 (351)	3.1 (1.1)	279 (232)	1.6 (1.3)	314 (176)	2.0 (1.1)
≥75, <80	611 (284)	3.0 (1.4)	304 (206)	1.6 (1.1)	356 (187)	2.1 (1.3)	551 (298)	3.0 (1.4)	304 (195)	1.6 (1.1)	356 (172)	2.1 (1.3)	631 (285)	3.1 (1.3)	304 (241)	1.8 (1.1)	355 (236)	2.4 (1.5)
*H*	0.396	2.753	0.206	1.566	1.475	3.019	1.698	1.984	1.907	1.780	0.146	0.803	1.357	2.486	2.784	1.509	1.325	2.882
*p*	0.941	0.431	0.977	0.667	0.688	0.389	0.637	0.576	0.592	0.619	0.986	0.849	0.716	0.478	0.426	0.680	0.723	0.410
BMI(kg/m^2^)																		
17.8 ~ 24.4	619 (262)	3.1 (1.3)^a^	299 (218)	1.9 (1.1)	333 (220)	2.0 (1.6)	596 (237)	3.0 (1.3)	316 (215)	2.0 (1.3)	364 (225)	2.4 (1.3)	621 (262)	3.1 (1.1)^a^	296 (200)	1.7 (1.3)	294 (173)	1.9 (1.4)
24.5 ~ 25.7	599 (264)	3.0 (1.3)^b^	316 (211)	1.7 (1.1)	371 (219)	2.1 (1.3)	587 (269)	3.0 (1.1)	314 (221)	1.6 (1.1)	371 (240)	2.0 (1.3)	593 (279)	3.1 (1.1)^a^	307 (229)	1.9 (1.3)	366 (211)	2.3 (1.6)
25.8 ~ 27.7	600 (339)	3.0 (1.4)^b^	316 (246)	1.7 (1.3)	346 (266)	2.0 (1.3)	589 (310)	3.0 (1.3)	316 (244)	1.6 (1.1)	330 (353)	2.1 (1.3)	614 (317)	3.3 (1.3)^b^	321 (277)	1.9 (1.3)	381 (201)	2.1 (1.6)
27.7 ~ 36.6	530 (287)	3.0 (1.1)^c^	300 (237)	1.6 (1.4)	317 (197)	1.9 (1.1)	540 (278)	3.0 (1.1)	299 (180)	1.6 (1.3)	343 (191)	1.9 (0.8)	530 (304)	3.0 (1.3)^a^	309 (251)	1.6 (1.4)	314 (197)	1.9 (1.4)
*H*	4.330	12.806	0.351	2.103	3.876	6.008	6.964	6.118	0.036	5.681	1.086	6.964	3.077	8.355	0.301	0.740	6.695	5.337
*p*	0.228	0.005^*^	0.950	0.551	0.275	0.111	0.073	0.106	0.998	0.128	0.780	0.073	0.380	0.039^*^	0.960	0.864	0.082	0.149

Values presented as median (quartile ranges) and compared using the Kruskal–Wallis H test. The four groups of BMI were divided according to the quartile range of BMI. ^*^Means that there was significant difference among participants in different cities/BMI groups, as the value of *p* was less than 0.05. ^a–e^The same symbol indicates that there were no statistically significant differences between the two groups; different symbols indicate that the differences were statistically significant between the two groups. BMI, body mass index (kg/m^2^). Fluid intake (mL).

**Table 5:** Description of urination behaviors with different characteristics.

	Total					Males					Females				
	n^a^	Day		Night			Day		Night		*n*^a^	Day		Night	
		Numbers^b^	>7 N (%)^c^	Numbers^b^	≥1 N (%)^d^	*n*^a^	Numbers^b^	>7 N (%)^c^	Numbers^b^	≥ 1 N (%)^d^		Numbers^b^	>7 N (%)^c^	Numbers^b^	≥1 N (%)^d^
Total	524	7.0 (2.4)	232 (44.3)	0.7 (0.9)	406 (77.5)	23	7.0 (2.4)	99 (42.5)	0.7 (0.7)	192 (82.4)	29	7.0 (2.6)	133 (45.7)	0.7 (1.0)	214 (73.5)
*Z*							0.810	0.542	1.449	5.827					
*p*							0.368	0.462	0.229	0.016^#^					
Cities															
Taiyuan	93	6.9 (2.8)^d^	39 (41.9)^c^	0.7 (0.9)^c^	74 (79.6)^c^	39	6.3 (3.0)^d^	14 (35.9)	0.7 (0.9)^c^	32 (82.1)	54	6.9 (2.9)^c^	25 (46.3)^c^	0.7 (0.9)^c^	42 (77.8)^c^
Jinan	96	6.4 (2.6)^c^	28 (29.2)^c^	0.7 (0.7)^c^	79 (82.3)^c^	48	6.5 (2.5)^c^	16 (33.3)	0.7 (0.7)^c^	39 (81.3)	48	6.3 (2.8)^d^	12 (25.0)^d^	0.7 (0.6)^c^	40 (83.3)^c^
Hefei	121	6.3 (2.3)^e^	40 (33.1)^d^	0.9 (0.4)^d^	110 (90.9)^d^	53	6.7 (2.0)^e^	19 (35.8)	0.9 (0.4)^d^	48 (90.6)	68	6.0 (2.5)^e^	21 (30.9)^e^	0.7 (0.6)^c^	62 (91.2)^d^
Nanchang	116	7.3 (3.0)^f^	63 (54.3)^c^	0.6 (1.0)^e^	84 (72.4)^e^	54	7.1 (3.3)^d^	27 (50.0)	0.7 (0.8)^c^	44 (81.5)	62	7.6 (2.5)^f^	36 (58.1)^f^	0.6 (1.0)^d^	40 (64.5)^c^
Guangzhou	98	7.5 (1.7)^e^	62 (63.3)^d^	0.3 (1.0)^f^	59 (60.2)^f^	39	7.3 (2.7)^e^	23 (59.0)	0.7 (1.0)^c^	29 (74.4)	59	7.7 (1.4)^f^	39 (66.1)^f^	0.1 (0.6)^e^	30 (50.8)^e^
*H*		44.337	34.318	31.362	32.483		13.784	8.880	4.407	4.255		31.753	28.028	34.938	31.492
*p*		<0.001^*^	<0.001^*^	<0.001^*^	<0.001^*^		<0.001^*^	0.064	<0.001^*^	0.373		<0.001^*^	<0.001^*^	<0.001^*^	<0.001^*^
Age (y)															
≥60, <65	104	6.9 (2.3)	39 (37.5)	0.4 (1.0)^c^	71 (68.3)	38	6.3 (1.8)	11 (28.9)	0.7 (0.8)	30 (78.9)	66	7.0 (2.3)	28 (42.4)	0.4 (1.0)^c^	41 (62.1)
≥65, <70	149	7.0 (2.6)	73 (49.0)	0.7 (0.9)^d^	119 (79.9)	45	6.9 (3.0)	22 (48.9)	0.9 (0.8)	40 (88.9)	10	7.0 (2.5)	51 (49.0)	0.7 (0.9)^d^	79 (76.0)
≥70, <75	155	7.0 (2.6)	69 (44.5)	0.9 (0.7)^e^	121 (78.1)	82	7.0 (2.3)	36 (43.9)	0.7 (0.9)	64 (78.0)	73	7.0 (2.9)	33 (45.2)	0.9 (0.6)^e^	57 (78.1)
≥75, <80	116	7.0 (2.6)	51 (44.0)	0.7 (0.7)^d^	95 (81.9)	68	7.0 (2.1)	30 (44.1)	0.7 (0.7)	58 (85.3)	48	6.7 (3.3)	21 (43.8)	0.7 (0.9)^d^	37 (77.1)
*H*		1.848	3.287	9.130	6.870		3.796	3.747	2.086	3.083		0.704	0.833	8.338	5.82
*p*		0.605	0.349	0.028^*^	0.076		0.284	0.290	0.555	0.379		0.872	0.842	0.040^*^	0.121
BMI (kg/m^2^)															
17.8 ~ 24.4	131	7.3 (2.6)^c^	70 (53.4)	0.7 (1.0)^c^	98 (74.8)^c^	54	7.3 (3.1)^c^	32 (59.3)^c^	0.8 (0.6)^c^	44 (81.5)	77	7.0 (2.4)	38 (49.4)	0.4 (1.0)^c^	54 (70.1)
24.5 ~ 25.7	131	7.0 (2.7)^d^	58 (44.3)	0.7 (0.7)^c^	107 (81.7)^d^	62	7.0 (2.5)^d^	27 (43.5)^d^	0.7 (0.9)^d^	52 (83.9)	69	7.0 (3.0)	31 (44.9)	0.9 (0.6)^d^	55 (79.1)
25.8 ~ 27.7	131	7.0 (2.3)^d^	56 (42.7)	0.9 (0.6)^d^	109 (83.2)^d^	68	6.7 (2.1)^e^	22 (32.4)^d^	0.9 (0.7)^e^	59 (86.8)	63	7.4 (2.7)	34 (54.0)	0.9 (0.6)^d^	50 (79.4)
27.7 ~ 36.6	131	6.7 (2.1)^e^	48 (36.6)	0.6 (1.0)^e^	92 (70.2)^e^	49	6.4 (2.4)^f^	18 (36.7)^e^	0.6 (0.8)^f^	37 (75.5)	82	6.9 (2.0)	30 (36.6)	0.5 (1.0)^c^	55 (67.1)
*H*		8.686	7.673	0.000	8.269		8.851	9.767	7.689	2.621		0.000	4.911	0.000	4.671
*p*		0.034^*^	0.053	0.002^*^	0.041^*^		0.031^*^	0.021^*^	0.053	0.454		0.312	0.178	0.009^*^	0.198

^a^Values presented as numbers of each group and compared using chi-squared test. ^b^Values presented as median (quartile ranges) and compared using the Kruskal–Wallis H test. ^c^Values means the number (percentages) of participants who have more than 7 urinations in a day. These were compared using the Kruskal–Wallis H test. ^d^ Values means the number (percentages) of participants who have urination at night (Once participants engaged in urination at night, they were included in the “≥ 1” group, otherwise they were not). These were compared using the Kruskal–Wallis H test. The four groups of BMI were divided according to the quartile range of BMI. ^#^Means that there was significant difference between males and females. ^*^Means that there was significant difference among participants in different cities/age groups/BMI groups, as the value of *p* was less than 0.05. ^c–g^The same symbol indicates that there were no statistically significant differences between the two groups; different symbols indicate that the differences were statistically significant between the two groups. BMI, body mass index (kg/m^2^).

During the study period, the average temperatures calculated in Jinan, Taiyuan, Nanchang, Hefei, and Guangzhou were 17°C, 13°C, 22°C, 19°C, and 26°C, respectively.

### 3.2 Fluid intake patterns of the participants with different characteristics

Among the 524 participants, the median daily TFI was 1,241 mL, as the median values for five cities were 1,401 mL, 1,219 mL, 1,164 mL, 1,369 mL, and 1,231 mL, respectively. The results showed that participants from Taiyuan had the highest median of TFI, while those from Hefei had the lowest one (*H* = 10.347, *p* < 0.05). Comparing the TFI of male participants and female participants, no significant differences were found between the participants in the two groups (*p* < 0.05), while the fluid intake of different types differed significantly (all *p* < 0.05). The percentage of participants who had not meet the recommendations of adequate fluid intake level in China was 73.3% (79.0% for male participants, 68.7% for female participants), based on a fluid intake of 1.7 L for male adults and 1.5 L for female adults by the Chinese Nutrition Society (72). Plain water was the main source of fluid intake, accounting for 69.9%. By comparing the results of five cities, it was found that the participants of Taiyuan had the highest intake levels of plain water and dairy products (1,049 mL, 217 mL, respectively). The participants of Jinan had the highest levels of tea intake (M = 114 mL). The elderly in all five cities had low intake levels of SSBs (Table 3).

Regarding BMI, dairy product intake between the total participants and female participants differed significantly between the four groups (*H* = 8.784, *p* < 0.05, *H* = 8.625, *p* < 0.05). Participants with normal BMI (17.8 ~ 24.4) had the highest dairy products intake level. The intake level of tea was significantly different among the four groups of male participants (*H* = 9.138, *p* < 0.05).

Few people consumed soybean milk, fruit/vegetable juices, and other beverages, and the intake levels were very small. Therefore, the data are not presented in the table (Table 3).

### 3.3 Daily fluid intake of participants with different characteristics in different time periods

The median TFIs of the participants in morning, afternoon, and evening were 594 mL, 305 mL, and 342 mL, respectively. There existed statistically significant differences in terms of fluid intake between the three periods (*H* = 619.948, *p* < 0.05). Significant differences were found in the frequency of fluid intake between the morning, afternoon, and evening periods (*H* = 660.417, *p* < 0.05). Among all participants, fluid intake and fluid intake frequency in the morning had the highest median values. The fluid intake in the morning differed significantly among the participants in the five cities (*H* = 14.540, *p* < 0.05). No significant differences were observed in terms of fluid intake in the afternoon and evening among the participants in the five cities (both *p* > 0.05). The fluid intake frequency of the participants of the five cities in three periods differed significantly (all *p* < 0.05). The participants with a BMI between 17.8 and 24.4 kg/m^2^ had the highest fluid intake frequency in the morning (M = 3.1) (Table 4).

### 3.4 Urination behaviors of participants with different characteristics

The median frequency of urination of all participants was 7.0 times during the day and 0.7 times in night. There was no statistically significant difference in the frequency of urination between male and female participants (7.0 times per day and 0.7 times per night for both). The frequency of urination differed significantly between participants from five cities (*H* = 44.337, *p* < 0.05) and among the BMI groups (*H* = 8.686, *p* < 0.05). Approximately 4.3% of the participants urinated more than seven times during the day, and 77.5% of participants urinated one time or more during the night across the five cities. For total and female participants, the percentage who urinated more than seven times during the day differed significantly between different cities (*H* = 34.318, *p* < 0.05; *H* = 28.028, *p* < 0.05). For female participants, there existed significant differences in the percentage who urinated more than seven times during the day between the four groups of BMI (*H* = 9.767, *p* < 0.05). The percentage of participants who urinated more than once during the night differed significantly between males and females (*H* = 5.827, *p* < 0.05) and between different cities (*H* = 32.483, *p* < 0.05) (Table 5).

### 3.5 Correlations between fluid intake behaviors and urination behaviors

Linear analysis showed that higher TFI, plain water intake, dairy products intake, and fluid intake frequency were significantly associated with higher urination frequency (*t* = 6.553, *p* < 0.05; *t* = 5.291, *p* < 0.05; *t* = 4.667, *p* < 0.05; *t* = 13.413, *p* < 0.05). Inatead, higher fluid intake per time was significantly associated with lower urination frequency (*t* = −3.562, *p* < 0.05) (Table 6).

**Table 6:** Correlations between urination frequency and fluid intake behaviors in 24 h.

Variables	Dependent variable	*F*	Unstandardized coefficient		Standardized coefficient	*t*	*p*
			*B*	*SE*	*β*		
TFI (mL)	Urination frequency	42.946	0.001	0.000	0.276	6.553	<0.001^*^
Plain water (mL)		27.994	0.001	0.000	0.226	5.291	<0.001^*^
Tea (mL)		0.012	4 × 10^−5^	0.000	0.005	0.110	0.912
Dairy products (mL)		21.778	0.003	0.001	0.200	4.667	<0.001^*^
Fluid intake frequency		179.899	0.453	0.034	0.506	13.413	<0.001^*^
Fluid intake per time		12.691	−0.006	0.002	−0.154	−3.562	<0.001^*^

Linear regression models were used to examine relationships between urination frequency and fluid intake behaviors. ^*^Means that there was statistically significant correlations, as the value of p was less than 0.05. TFI: total fluid intake.

The correlations of urination frequency, urination in night and fluid intake behaviors were also analyzed in the present study. The results showed that there exist correlations between urination frequency and TFI, fluid intake in afternoon, fluid intake frequency in afternoon, and fluid intake in night, and fluid intake frequency in night (all *p* < 0.05). Correlations were found between urination in night and TFI, fluid intake frequency, fluid intake in night, and fluid intake frequency in night (all *p* < 0.05) (Table 7).

**Table 7:** Correlations between fluid intake and urination behavior at night.

	TFI		Fluid intake frequency		Fluid intake in afternoon		Fluid intake frequency in afternoon		Fluid intake in night		Fluid intake frequency in night	
	*r*	*p*	*r*	*p*	*r*	*p*	*r*	*p*	*r*	*p*	*r*	*p*
Urination frequency	17.084	<0.001^*^	27.071	<0.001^*^	4.630	0.032^*^	6.008	0.015^*^	34.242	<0.001^*^	67.360	<0.001^*^
≥1 N^a^	0.114	0.009^*^	0.091	0.038^*^	0.069	0.113	0.041	0.354	0.146	<0.001^*^	0.331	<0.001^*^

Spearman’s correlation coefficients were used to analyze the correlations among urination frequency and fluid intake behaviors. Point biserial correlations were used to analyze the correlations among fluid intake behaviors and night urination. ^a^Value means the participants who have urination at night (Once participants engaged in urination at night, they were included in the “≥1” group, otherwise they were not). ^*^Means that there were statistically significant correlations, as the value of *p* was less than 0.05. TFI: total fluid intake.

## 4 Discussion

In the present study, over half of the participants did not meet the recommended amount of adequate fluid intake in China (excluding water from food, 1.7 L/d for male adults and 1.5 L/d for female adults). Statistical differences between the participants from different cities exist. This may be influenced by the inhabitants’ habit of drinking soup or eating rice soaked in soup in Guangzhou, which may increase the proportion of water intake in food sources, leading to a decrease in fluid intake levels (73). In addition, considering the temperature differences of five cities (up to even 13°C), the fluid intake behaviors may be influenced by water loss caused by sweating and heat dissipation. This was similar to the result of a study showing that fluid intake from beverages was positively associated with temperature. The results obtained in this study suggest that attention should be paid to promoting knowledge and skills related to fluid intake of the elderly through health education. A survey conducted on 413 middle-aged and elderly people aged 50 ~ 75 years in Jinan, China, found that the median TFI of people over 60 years old ranged from 950 to 1,050 mL. This was lower than the median TFI of the elderly in Jinan city (1,219 mL) in our study. This may be influenced by different study methods, as a 24-h recording method for 7 consecutive days was used in our study, while recording across four consecutive days was used in the previous one (34). The water requirements of the elderly were affected by many factors, including micro-environment (temperature, humidity, wind speed), individual health status, and physical activity (21, 22). This study found that plain water was the main source of TFI for participants, accounting for 69.9%. This was similar to the results gathered among adults in different stages in China (74–76). Dairy products were the second-highest contributors to fluid intake among the participants. However, the milk intake level (M = 193 mL) was much lower than the recommended amount by the Chinese Nutrition Society (300 mL) (22). Compared with previous studies conducted in China, the elderly had higher tea intake than young adults and pregnant women (74, 75). It should be noted that there existed differences in fluid intake patterns between the five cities, which suggested that health education and strategies suiting local conditions should be conducted. Besides, it was revealed that the fluid intake level in the morning was much higher than the levels in afternoon and evening (Table 4). This may be due to the Chinese dietary habits, as breakfast often goes with congee (a Chinese rice gruel eaten for breakfast).

The frequency of urination in healthy adults is 4 ~ 8 times per day (77). Frequent urination was defined as urinating more than seven times during the day (70). Nocturia was defined as urinating more than once at night to urinate, according to the International Continence Society report on urinary function (71). In our study, the percentage of participants urinating more than seven times per day was 44.3%, and the incidence of nocturia was 77.5%. Compared with earlier studies conducted in China, the incidence of frequent urination and nocturia in our study is higher than the results among female senior citizens (25, 40%) (78) and college students (25, 40%) (79). The results were consistent with those observed in previous studies, which have revealed that frequent urination and nocturia have a higher incidence in the elderly. A study on the hospitalized elderly in Rome showed that the incidence of frequent urination was 67% (80). This may possibly be due to the decrease in bladder storage capacity with age, resulting in an increase in urination frequency. Furthermore, it is worth noting that frequent urination and nocturia are manifestations of kidney diseases such as chronic kidney disease and overactive bladder and may also be caused by mental stimulation or excessive drinking water (81). A study conducted on the elderly in Sweden showed that the incidence of nocturia was approximately 70% (82). Another study suggests that urinating at night in the elderly is a normal feature of physical decline (83). For the elderly, frequent urination can lead to multiple visits to the toilet. Urination at night influences sleep quality and increases the risk of falls and fractures (84).

It should be noted that the result of our study revealed that correlations exist between fluid intake frequency and nocturia. This suggests that nocturia in the elderly can be improved by limiting their fluid intake behaviors before bedtime, including volume and frequency. Thus, the sleep quality of the elderly could be improved in this way. In recent years, many studies on fluid intake and urination have been carried out in many countries on individuals of different ages. Some of them explored the effect of fluid intake on the urinary system in order to seek economic, safe, and simple approaches for the prevention or treatment of urinary system diseases (85, 86). The results in our present study were consistent with the results obtained from a controlled trial study on the elderly (87). The studies modified their lifestyles and restricted fluid intake to seek measurements in treating nocturia. The results showed that mean nocturnal urination times and volume decreased significantly from 3.6 to 2.7 times and from 923 to 768 mL, respectively. Another study revealed that guidance on water intake had an effect on the degree of nocturia, which concluded that fluid restriction should be performed not only in the evening but also during daytime (88). However, few studies found that there was no clear association between fluid intake and nocturia (89). The possible reason may be that the subjects were patients with urinary system disorders, and the urine metabolism had been disturbed. A previous study collected short-term urination, which was separated within 24 h, revealed that there were daily fluctuations in urine production (90). Influenced by the circadian pattern of arginine vasopressin release, urine volume was lower overnight, throughout the morning, and in the evening before going to sleep (91). These physiological factors should be considered when developing fluid intake strategies to improve urination behaviors. In addition, urine osmolality was always chosen as the indicator to evaluate hydration status when exploring the influence of fluid intake on hydration status. However the time period of urine collection and testing is worth considering as an influencing factor.

A previous study found that participants’ hydration status may be closely correlated with their fluid intake, urination behavior, and urine indicators. Research conducted on young people in China also revealed similar results (77, 92). Therefore, it is of great importance to explore suitable urine indicators to evaluate hydration status. Considering climate factors, fluid intake surveys should be conducted among the elderly of different age groups in other cities, which could represent seven major geographical divisions in China in the future. Daily losses of sweat, urine, and respiratory water, as well as the sensation of thirst caused by fluid consumption, can lead to a response to the physiological regulation of total body water. From the available literature and clinical findings, it is suggested that increasing fluid intake may play a potential role in altering the concentration of biomarkers in plasma, such as copeptin (93). Previous studies have indicated that individuals with habitually higher fluid intake have higher 24 h urine volume, higher urination frequency and higher urine concentration (94, 95). According to the above results, meeting the recommended amount of adequate fluid intake and allocating the appropriate frequency are challenges to the fluid intake literacy concerning the elderly.

Regarding the strengths of this study, to our knowledge, it was the first to investigate fluid intake patterns and urination behaviors among healthy elderly people in China. In the present study, the fluid intake patterns of the participants were investigated in detail, including different fluid types, frequency, and time. Moreover, we assessed the influence of fluid intake on urination behaviors at night so as to develop measures from the perspective of forming scientific water intake behaviors to improve the sleep quality of the elderly. In addition, a real-time recording for 7 consecutive days was used in this study to investigate urination behaviors. This could not only get more detailed, accurate, and dynamic data but also avoid the recall bias that commonly existed in retrospective investigations. Finally, we explored the association between fluid intake behaviors and urination behaviors in this study. This could reveal urination behaviors’ possibility of being indicators in evaluating fluid behaviors and hydration status. Despite the advantages mentioned above, the study did have some limitations. It is rather remarkable that only fluid intake levels were examined in the current study, as the levels of water obtained from food were not investigated. However, our previous research has shown that this was an important component of total water intake, which accounts for 50.7 to 51.5% (96). Participants with different TFI levels may have differences in water intake levels from food. Hence, i great importance is attached to conduct research on the dietary intake of participants to obtain the total water intake value. In addition, a previous study demonstrated that 24 h urine volume could be affected by fluid intake patterns, while the data were not collected in our study. Another concern about the findings was that the five cities in this study were not well-distributed, and the fluid intake levels of their inhabitants may be seasonally affected. As a result, this may lead to a limitation in the comprehensiveness of the time-point data obtained from this cross-sectional survey (97). Therefore, fluid intake research conducted across different times and different geographical regions in China should be considered. In this survey, the urine color of the participants was not explored and analyzed through objective and accurate measurements. Previous studies conducted on different participants indicated that differences in urine color were related to dehydration and hydration status (98–100). From these observations, great importance is attached to further exploring whether urine color can be an indicator of dehydration. In addition, blood and urine biomarkers were not tested in the present study. Hence, it is hard to assess the hydration status of the elderly so as to further explore the relationship between their fluid intake level and dehydration. In future studies, urine biomarkers, especially urine-specific gravity, should be tested to identify dehydration accurately and to explore its role in evaluating hydration status.

## 5 Conclusion

The TFI was inadequate for the elderly, with 73.3% of participants under the recommended amount of adequate fluid intake in China. The elderly exhibited several unhealthy urination behaviors, including urination at night. Participants with lower fluid intake levels had lower urination frequency, and participants with higher fluid intake and frequency experienced incidence of nocturia. Age and BMI were not the main influencing factors for fluid intake and urination behaviors. The results can contribute to strategies for promoting healthy fluid intake and urination behaviors, further improving the health of the elderly. Further studies for nationally representative data with more detailed detection of fluid intake, urine biomarkers, and hydration status are necessary to effectively analyze their impact on health for the elderly.

## Data availability statement

The original contributions presented in the study are included in the article/supplementary material, further inquiries can be directed to the corresponding author.

## Ethics statement

The studies involving humans were approved by the Ethical Review Committee of Peking University, identification code IRB 00001052-16071. The studies were conducted in accordance with the local legislation and institutional requirements. The participants provided their written informed consent to participate in this study.

## Author contributions

YS: Conceptualization, Data curation, Formal analysis, Writing – original draft, Writing – review & editing. YZ: Data curation, Formal analysis, Investigation, Writing – review & editing. YL: Resources, Writing – review & editing. JZ: Data curation, Investigation, Methodology, Project administration, Writing – review & editing. JL: Investigation, Software, Writing – review & editing. XW: Investigation, Resources, Software, Writing – review & editing. NZ: Data curation, Funding acquisition, Investigation, Methodology, Project administration, Supervision, Validation, Visualization, Writing – review & editing. GM: Funding acquisition, Methodology, Project administration, Supervision, Validation, Visualization, Writing – review & editing.
